# Evolution clinique de la myasthénie auto-immune en Neurologie à Antananarivo, Madagascar

**DOI:** 10.11604/pamj.2020.37.304.18733

**Published:** 2020-12-02

**Authors:** Santatra Ratsitohara Razafindrasata, Julien Razafimahefa, Glorien Jemissair Lemahafaka, Noël Zodaly, Alain Djacoba Tehindrazanarivelo

**Affiliations:** 1 Service de Neurologie, Hôpital Joseph Raseta Befelatanana, Antananarivo, Madagascar

**Keywords:** Evolution, Madagascar, myasthénie auto-immune, Clinical course, Madagascar, myasthenia gravis

## Abstract

**Introduction:**

la myasthénie auto-immune est une maladie rare, agissant au niveau de la jonction neuromusculaire. Elle peut engager le pronostic vital surtout lors d´une atteinte respiratoire. La rémission complète est possible sous traitement. Nos objectifs étaient de décrire l'évolution clinique sous traitement de nos patients myasthénique pour améliorer ultérieurement notre prise en charge et pour obtenir une première base de données malgaches.

**Méthodes:**

une étude rétrospective descriptive fut menée au sein du Service de Neurologie de l´Hôpital Universitaire Befelatanana entre janvier 2010 et décembre 2017, incluant tout patient diagnostiqué comme myasthénie auto-immune après un test à la prostigmine positif.

**Résultats:**

vingt-cinq patients (0,42%) sur 5814 hospitalisés étaient inclus. Seule l´évolution de 16 patients était connue (64%) dont 14 étaient sous traitement médical et 3 d´entre eux avaient une thymectomie. La durée moyenne de suivi était de 24 mois. Parmi les patients traités, 8 s´étaient améliorés, 2 étaient décédés. Chez les patients thymectomisés, 2 s´étaient améliorés, 1 était décédé.

**Conclusion:**

la myasthénie reste une pathologie rare, grave, mais l´amélioration sous traitement est possible même en absence d´un plateau technique adéquat pour sa prise en charge à Madagascar. En ce moment, un projet avec l´association des patients myasthéniques à Madagascar est en cours pour pouvoir bénéficier des appareils respiratoires utiles surtout lors des crises myasthéniques.

## Abstract (en)

**Introduction:**

myasthenia gravis (MG) is a rare disease affecting the neuromuscular junction. It can lead to a life-threatening condition, especially when it is associated with respiratory failure. Full remission is possible with treatment. Our aims are to describe the clinical course of patients with MG with under treatment, in order to further improve management and to implement a database of patients living in Madagascar.

**Methods:**

we conducted a descriptive retrospective study in the Department of Neurology at the Befelatanana Antananarivo University Hospital Center between January 2010 and December 2017. This study involved all patients diagnosed with MG based on positive prostigmin test.

**Results:**

among the 5814 hospitalized patients, 25 (0,42%) were included. Only 16 patients were on follow-up (64%) of whom 14 were receiving medical treatment. Three of them had undergone thymectomy. The mean follow-up period was 24 months. Among treated patients, 8 improved and 2 died. Among thymectomized patients, 2 improved and 1 died.

**Conclusion:**

myasthenia gravis is a rare but serious disease. Improvement is possible with treatment even in the absence of adequate technical equipment for its management in Madagascar. Currently, a campaign in partnership with Myasthenia Gravis Association in Madagascar is underway for breathing devices useful especially when myasthenic crises occur.

## Introduction

La myasthénie auto-immune ou myasthenia gravis (MG) est une maladie auto-immune caractérisée par un bloc de la transmission de l´influx nerveux au niveau de la jonction neuromusculaire en post-synaptique, secondaire à la production d´autoanticorps dirigés principalement contre les récepteurs à l´acétylcholine (RAch). Elle se traduit par la fatigabilité de la musculature striée [1]. La MG est une maladie rare, avec 8 à 10 cas par million d´habitants par an, mais elle existe partout dans le monde [2-8]. Elle est grave car peut engager le pronostic vital surtout lors d´une atteinte des muscles respiratoires c´est-à-dire lors des crises myasthéniques [1]. La prise en charge symptomatique des crises repose sur l´utilisation des immunoglobulines et des échanges plasmatiques. Les corticoïdes et les immunosuppresseurs constituent les traitements de fond de la MG [9]. La rémission est possible sous traitement. A Madagascar, peu d´études ont été publiées sur la myasthénie. Notre objectif était de décrire l´évolution clinique sous traitement de nos patients myasthéniques afin d´améliorer notre prise en charge et d´obtenir une première base de données.

## Méthodes

Il s´agit d´une étude rétrospective descriptive, au sein du Service de Neurologie de l´Hôpital Universitaire Befelatanana, allant de janvier 2010 jusqu´en décembre 2017. L´évènement attendu était soit le décès, la persistance des signes, l´aggravation des signes, l´amélioration des signes, la rémission clinique. Nous avons inclus tout patient diagnostiqué comme myasthenia gravis établit par le test à la prostigmine positif avec l´aide du score myasthénique [1,10]. Nous avons étudié les caractères démographiques à savoir l´âge (inférieur ou égal à 40 ans et supérieur à 40 ans) et le genre; les caractéristiques clinico-radiologique de la myasthénie dont le type de myasthénie, la présence ou non d´un thymome ou d´une tumeur de la glande thymique ; la prise en charge médicamenteuse et/ou chirurgicale (thymectomie); ainsi que son évolution clinique incluant le décès.

Pour classifier la myasthénie, nous avons utilisé l´ancienne classification d´Ossermann qui permet de classer la myasthénie, débutant par une myasthénie localisée stade I, puis une myasthénie généralisée minime sans signes bulbaires stade IIa, ensuite une myasthénie généralisée modérée avec signes bulbaires stade IIb, par la suite une myasthénie généralisée sévère à début aigu avec signes respiratoires stade III, enfin une myasthénie évoluée grave potentiellement mortelle stade IV [8]. L´analyse statistique des données était effectuée par Epi-info 7.

## Résultats

Sur 5814 patients hospitalisés en neurologie, nous avons eu 25 nouveaux cas de myasthénie soit 0,42%. Après les critères d´inclusion et d´exclusion, nous avons retenus 16 patients (0,27%). Tous les patients retenus ont eu un suivi moyen de 24 mois. Nos patients sont d´âge moyen de 40 ans à prédominance féminine avec un sex-ratio de 0,45. La myasthénie stade III, une myasthénie généralisée sévère avec atteinte respiratoire était la plus fréquente (n=6 soit 37,5%). Trois patients (18,75%) avaient un thymome, ils étaient tous de stade A selon la classification OMS 2004. Seuls 2 de nos patients (12,5%) n´ont pas pu bénéficier d´un traitement médicamenteux faute de moyen financier.

Le traitement médicamenteux était basé sur l´utilisation d´un traitement anticholinestérasique (pyridostigmine ou mestinon*). Tous les patients ayant un thymome ont pu bénéficier d´une thymectomie (Tableau 1). Parmi les patients avec traitement, une amélioration à plus de la moitié des cas a été retrouvée, avec 2 décès (14%) survenant à moins de 6 mois du diagnostic dans un tableau de détresse respiratoire aiguë d´une crise myasthénique. Parmi les patients thymectomisés, deux s´étaient améliorés et un décédé (Tableau 2).

**Tableau 1:** répartition démographique, clinico-radiologique et thérapeutique

Patients (n=16)		n	%
**Age**			
	≤40 ans	4	25
	≤40 ans	12	75
**Genre**			
	Masculin	5	31,25
	Féminin	11	68,75
**Type myasthénie**			
	Stade I	2	12,5
	Stade IIa	5	31,25
	Stade Iib	3	18,75
	Stade III	6	37,5
**Thymome**		3	18,75
**Thymectomie**		3	100
**Traitement médicamenteux**		14	87,5
	Mestinon	9	64,29
	Mestinon et corticoïde	3	21,43
	Mestinon et azathioprine	1	7,14
	Mestinon, corticoïde et azathioprine	1	7,14

**Tableau 2:** répartition des patients selon leur évolution clinique

Patients	Avec traitement (n=14)		Sans traitement (n=2)		Thymectomisés (n=3)	
	n	%	n	%	n	%
Décès	2	14,29	1	50	1	33,34
Amélioration	8	57,14	0	0	2	66,66
Persistance symptôme	3	21,43	0	0	0	0
Aggravation	1	7,14	1	50	0	0
Rémission	0	0	0	0	0	0

## Discussion

Dans nos résultats, nous avons trouvé des améliorations des manifestations cliniques de la myasthénie chez la plupart de nos patients traités, dont le principal traitement était basé sur la prise d´anticholinestérasique (pyridostigmine ou mestinon*), qu´on utilise en première intention dans notre pratique quotidienne pour le traitement symptomatique dans le service. Cette étude nous a permis d´une part d´avoir une première base de données sur la myasthénie même si elle n´est pas représentative à l´échelle nationale. Il s´agit d´une des rares études sur la myasthénie effectuée à Madagascar en ayant vérifié sur les thèses en ligne malgache, Google et Google Scholar, PubMed. D´autre part, elle nous a permis d'actualiser nos connaissances sur les avancées actuelles de la myasthénie auto-immune. Cependant, la contrainte majeure est le contexte socio-économique, c´est-à-dire l´inaccessibilité des examens sérologiques par nos patients, l´absence d´un examen électroneuromyographique (ENMG) pour confirmer le diagnostic [1,11]. Ainsi, le diagnostic de myasthénie a été posé cliniquement après le test positif à la prostigmine.

Concernant les caractères démographiques, elle est similaire à une étude de Fall M *et al*. au Sénégal [4] qui retrouvait 5 cas en 3ans, dont l´âge variait entre 25 à 38ans, avec une prédominance féminine. Ces résultats sont conforment aux données de la littérature sur la rareté de la myasthénie, et la prédominance féminine de la myasthénie avant l´âge de 40 ans [1,12]. En effet, les œstrogènes seraient parmi les responsables du développement des maladies auto-immunes y compris la myasthénie, et favoriseraient leur prévalence chez les femmes avant la ménopause selon les résultats d´une étude de Dragin N *et al*. [1,13].

Nous avons stadifié la myasthénie à partir de l´ancienne classification d´Ossermann qui est encore utilisé dans d´autres études comme celle de Muhammed J *et al*. [8] et Aydin Y *et al*. [14]. Dans ces deux études, cette classification était nécessaire pour une évaluation pré-opératoire avant la réalisation d´une thymectomie. Elle est plutôt facile à utiliser et à retenir dans la pratique quotidienne. Mais la classification *Myasthenia Gravis Foundation of America (MGFA)* est la classification la plus utilisée actuellement dans les études sur la myasthénie [15]. La forme généralisée avec atteinte respiratoire était la plus fréquemment rencontrée. Cette forme sévère est liée au fait que les patients n'étaient vus par les neurologues qu´au stade tardif de la maladie. Dans 50% des cas, les manifestations oculaires peuvent être inaugurales de la myasthénie [1], les patients ont tendance à aller consulter chez les ophtalmologues au début. Après 2 ans, la maladie se généralise chez 80 à 90% des patients par atteinte des muscles pharyngolaryngés, muscles des membres voire muscles respiratoires [1].

A Madagascar, comme dans d´autres pays africains, les traitements disponibles et accessibles sont les anticholinestérasiques (pyridostigmine ou mestinon*), les corticoïdes (prednisone), et la thymectomie. Concernant les immunosuppresseurs, il est possible de les importer de l´extérieur en cas de nécessité. Les immunoglobulines sont d´accessibilité limitée pour les patients. Et les échanges plasmatiques ne sont pas encore disponibles dans les hôpitaux de Madagascar. En effet, en cas de crises myasthéniques, nous avons seulement recours à la réanimation avec assistance respiratoire alors que la principale cause de nos décès est la détresse respiratoire aiguë lors d´une crise myasthénique.

Néanmoins, nous avons trouvé une amélioration sous traitement médicamenteux, notamment sous anticholinestérasique, même avec une atteinte bulbaire et respiratoire. Ces résultats rejoignent celui de l´étude de Fall M *et al*. au Sénégal [4]. Les anticholinestérasiques restent la base du traitement symptomatique [4,9,10]. Nous avons constaté également une amélioration de tous les patients qui ont bénéficié d´une thymectomie. Plusieurs auteurs ont démontré l´efficacité de la thymectomie avec ou sans anomalie thymique comme dans l´essai MGTX [16], ou encore dans l´étude de Kaufman AJ *et al*. [17] ou celle de Taioli E *et al*. [18]. L´essai MGTX, un essai randomisé, contrôlé, a évalué l´efficacité de l´ablation chirurgicale du thymus (ou thymectomie) associée à une prise de prednisone en comparaison à celle d´une prise de prednisone seule chez des personnes atteintes de myasthénie non thymomateuse. Il a été mené par un groupe d´experts de la myasthénie dans 36 centres dans le monde entre 2006 et 2012. Les résultats ont montré que la thymectomie entraine à 3 ans un bénéfice clinique significatif ainsi que le recours à une dose de prednisone plus faible. L´association de l´azathioprine au traitement par prednisone s´est avérée plus souvent nécessaire chez les participants n´ayant pas subi de thymectomie. Cet essai est le premier à avoir évalué les effets de la thymectomie de façon randomisée et contrôlée et à apporter la preuve de l´effet bénéfique de cette procédure chez les personnes atteintes de myasthénie non thymomateuse liée à des autoanticorps anti-RACh [16].

En ce qui concerne le décès, nous avons eu 14% de décès à moins de six mois du diagnostic. Selon Hansen *et al*. [3], le décès survient surtout durant les 5 premières années du diagnostic. Le décès peut dans la plupart des cas être associé à d´autres étiologies. En premier lieu, en rapport avec la sévérité de la myasthénie au moment du diagnostic (crise myasthénique) surtout si associé à d´autres conditions réduisant l´autonomie comme la malnutrition, la fracture osseuse, ou la prise de traitement immunosuppresseur. En second lieu, liés aux effets indésirables des médicaments comme les crises cholinergiques, le sepsis, l´ostéoporose, les lymphomes ou autres types de cancers. En dernier lieu, étiologies liées à l´association de la MG avec d´autres maladies auto-immunes [5]. Nous envisageons de continuer l´étude en essayons de décrire un peu plus la mortalité et de déterminer la survie de nos patients myasthéniques.

## Conclusion

La myasthénie reste un grand défi à Madagascar même dans le monde. A l´issue de cette étude, il s´agit d´une pathologie rare, grave mais l´amélioration sous traitement même sous anti-cholinestérase qui est le seul disponible à Madagascar, est possible, même en absence de plateaux techniques adéquat. Par l´aide de l´association des patients myasthéniques à Madagascar, il existe un projet qui est en cours, nous permettant de bénéficier des appareils respiratoires utiles surtout pour la prise en charge des crises myasthéniques principale cause de décès des patients myasthéniques.

### Etat des connaissances sur le sujet

- Le diagnostic de myasthénie est confirmé par l´électroneuromyogramme (ENMG);
- La myasthénie est une maladie qui existe partout dans le monde.

### Contribution de notre étude à la connaissance

- L´utilisation du test à la prostigmine pour confirmer le diagnostic de myasthénie, très utile pour les autres pays à faible revenu qui n´ont pas d´ENMG;
- La première base de données malgache sur la myasthénie.

## Remerciements

Nous remercions tous ceux qui ont participé de loin ou de près à la réalisation de l´étude.
